# Intravital Imaging of Candida albicans Identifies Differential *In Vitro* and *In Vivo* Filamentation Phenotypes for Transcription Factor Deletion Mutants

**DOI:** 10.1128/mSphere.00436-21

**Published:** 2021-06-23

**Authors:** Rohan S. Wakade, Manning Huang, Aaron P. Mitchell, Melanie Wellington, Damian J. Krysan

**Affiliations:** a Department of Pediatrics, Carver College of Medicine, University of Iowa, Iowa City, Iowa, USA; b Department of Biological Sciences, Carnegie Mellon University, Pittsburgh, Pennsylvania, USA; c Department of Microbiology, University of Georgia, Athens, Georgia, USA; d Department of Microbiology and Immunology, Carver College of Medicine, University of Iowa, Iowa City, Iowa, USA; University of Texas Health Science Center

**Keywords:** *Candida albicans*, hyphae, intravital imaging, transcription factors

## Abstract

Candida albicans is an important cause of human fungal infections. A widely studied virulence trait of C. albicans is its ability to undergo filamentation to hyphae and pseudohyphae. Although yeast, pseudohyphae, and hyphae are present in pathological samples of infected mammalian tissue, it has been challenging to characterize the role of regulatory networks and specific genes during *in vivo* filamentation. In addition, the phenotypic heterogeneity of C. albicans clinical isolates is becoming increasingly recognized, while correlating this heterogeneity with pathogenesis remains an important goal. Here, we describe the use of an intravital imaging approach to characterize C. albicans filamentation in a mammalian model of infection by taking advantage of the translucence of mouse pinna (ears). Using this model, we have found that the *in vitro* and *in vivo* filamentation phenotypes of different C. albicans isolates can vary significantly, particularly when *in vivo* filamentation is compared to solid agar-based assays. We also show that the well-characterized transcriptional regulators Efg1 and Brg1 appear to play important roles both *in vivo* and *in vitro*. In contrast, Ume6 is much more important *in vitro* than *in vivo*. Finally, strains that are dependent on Bcr1 for *in vitro* filamentation are able to form filaments *in vivo* in its absence. This intravital imaging approach provides a new approach to the systematic characterization of this important virulence trait during mammalian infection. Our initial studies provide support for the notion that the regulation and initiation of C. albicans filamentation *in vivo* is distinct from *in vitro* induction.

**IMPORTANCE** Candida albicans is one of the most common causes of fungal infections in humans. C. albicans undergoes a transition from a round yeast form to a filamentous form during infection, which is critical for its ability to cause disease. Although this transition has been studied in the laboratory for years, methods to do so in an animal model of infection have been limited. We have developed a microscopy method to visualize fluorescently labeled C. albicans undergoing this transition in the subcutaneous tissue of mice. Our studies indicate that the regulation of C. albicans filamentation during infection is distinct from that observed in laboratory conditions.

## INTRODUCTION

Microbial virulence traits and factors are frequently studied using *in vitro* experimental systems, particularly when the goal is to probe detailed molecular mechanisms of pathogenesis. The premise of such experiments is based on a correlation between the *in vitro* observations and the events that occur during infection of the host; this assumption is frequently quite reasonable but also can be experimentally challenging to verify. Here, we describe the use of a novel intravital imaging approach to characterize the *in vivo* ability of Candida albicans to transition from yeast to filamentous morphology, a key virulence trait in this important, highly prevalent human fungal pathogen (1).

Candida albicans is one of the most common human fungal pathogens and causes both superficial mucosal infections, as well as invasive infections of organs such as liver, spleen, kidney, and brain. C. albicans undergoes characteristic morphologic transitions between round yeast and filamentous hyphae and pseudohyphae (2). Histopathologic analyses indicate that all three morphologic forms of C. albicans are generally present within infected anatomic sites. The transcriptional regulation of C. albicans filamentation has been the subject of extensive study and has led to the identification of transcription factors (TFs) that play roles in this morphogenetic transition (3, 4). Based on the study of three key hyphae-associated TFs (*EFG1*, *BRG1*, and *UME6*, along with the biofilm regulator Bcr1) in the standard reference strain SC5314 and four different clinical isolates of C. albicans, Huang et al. found that the transcriptional circuitry regulating *in vitro* biofilm formation and filamentation varied significantly among the strains (5).

We were interested in determining the roles of these TFs during *in vivo* filamentation. It is clear from a variety of studies that the ability of a given C. albicans mutant to undergo filamentation *in vitro* can vary with the specific *in vitro* inducing stimulus (6, 7). The existence of condition-dependent filamentation programs was nicely demonstrated by the systematic analysis reported by Azadmanesh et al. (6). We hypothesized that filamentation during mammalian infection may have characteristics that are distinct from *in vitro* filamentation. Currently, there are limited approaches to directly studying C. albicans morphologic transitions during infection. Histologic analyses of infected organs can provide information about filamentation. However, quantitative analysis is difficult because hyphae sectioned perpendicular to the long axis can appear as yeast. The zebrafish model has recently been used to advantage to characterize filamentation *in vivo* and provided a number of insights into the roles of both yeast and filaments during infection (8). For mammalian models, Witchley et al. recently reported a fluorescence *in situ* hybridization (FISH)-based approach that is applicable to the quantitative characterization of C. albicans filamentation during the colonization of the murine gastrointestinal (GI) tract (9).

**FIG 1:**
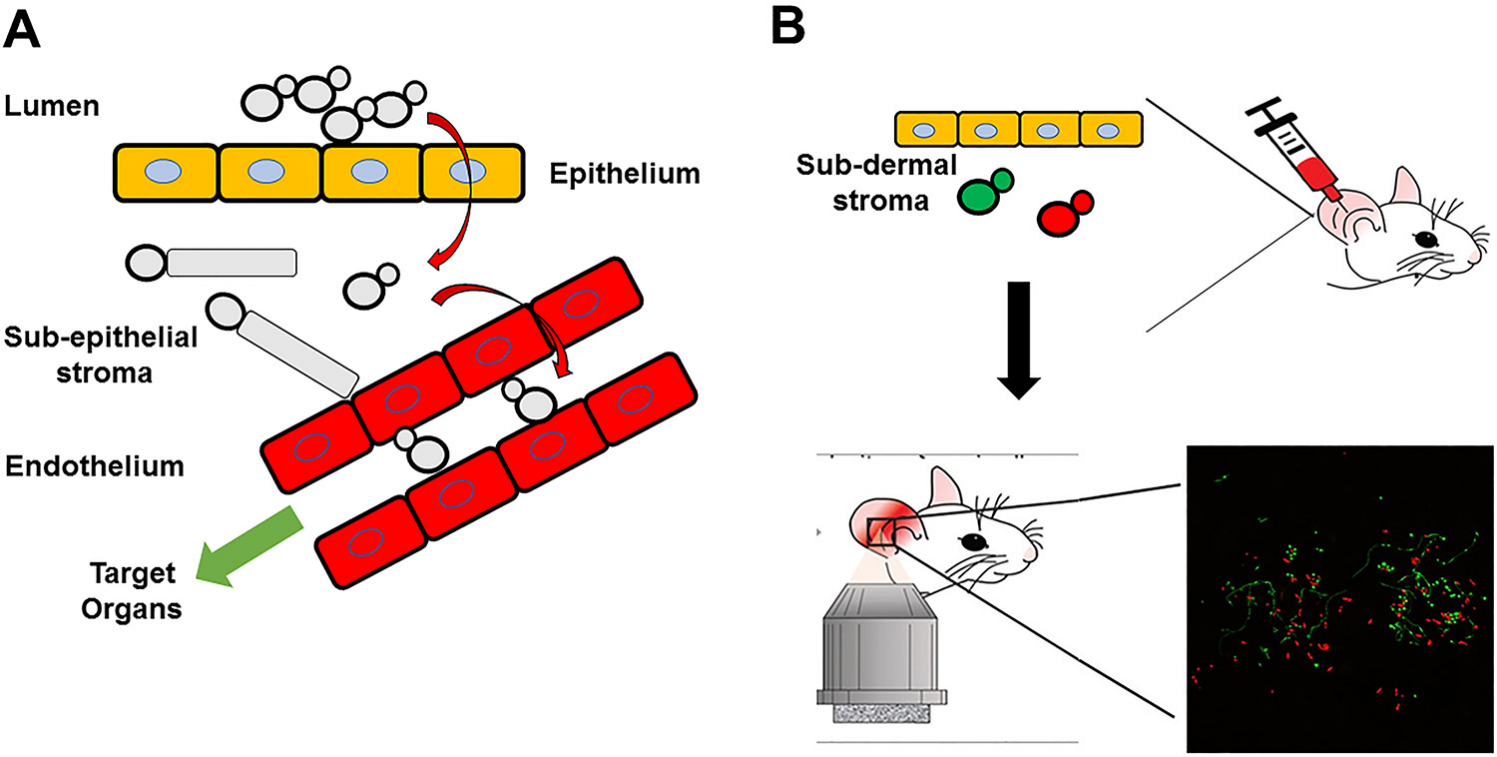
Premise and schematic of intravital imaging of Candida albicans in ear tissue of mice. (A) Model of translocation and dissemination of C. albicans. (B) Schematic representing injection of orthogonally labeled fluorescent C. albicans and imaging with confocal microscope.

Here, we report a novel intravital microscopy approach that has allowed us to characterize the C. albicans yeast-to-filament transition in a mouse model of infection (10). C. albicans is both a commensal colonizer of the human gastrointestinal (GI) tract and a cause of invasive infections (1). A well-accepted model for the transition from commensal colonization to pathogenic dissemination (11) begins with C. albicans breeching the epithelial cell layer of a mucosal tissue such as the oral cavity or the GI tract to invade the subdermal/submucosal stroma (Fig. 1A). Next, the fungus gains access to the vascular system by traversing the endothelial cells of blood vessels and, ultimately, disseminates to target organs such as the kidney, liver, spleen, and brain. A classic study by Saville et al. using a tetracycline-responsive allele of the repressor of filamentation *NRG1* indicated that yeast-locked strains established infection by dissemination through the bloodstream but did not cause disease until *NRG1* expression was repressed and filamentation occurred (12). This study clearly showed that morphology plays distinct roles in C. albicans pathogenesis. Similarly, the interactions of C. albicans with epithelial cells, endothelium, and target organs have been studied extensively (13).

In contrast, little is known about the interactions of C. albicans with subepithelial tissue and stroma. To study this stage of infection and to characterize *in vivo* filamentation of C. albicans, we adapted an intravital imaging method developed in our lab in which fluorescently labeled C. albicans are directly injected into the ear of mice (10) and observed using confocal microscopy (Fig. 1B). Using this approach, we demonstrate that: (i) the correlation between *in vitro* and *in vivo* filamentation phenotypes is dependent on the specific *in vitro* induction stimuli and (ii) the transcriptional regulation of *in vivo* filamentation is distinct from *in vitro* filamentation.

## RESULTS

### Correlation between *in vitro* and *in vivo* filamentation phenotypes varies with the specific *in vitro* induction stimuli.

**FIG 2:**
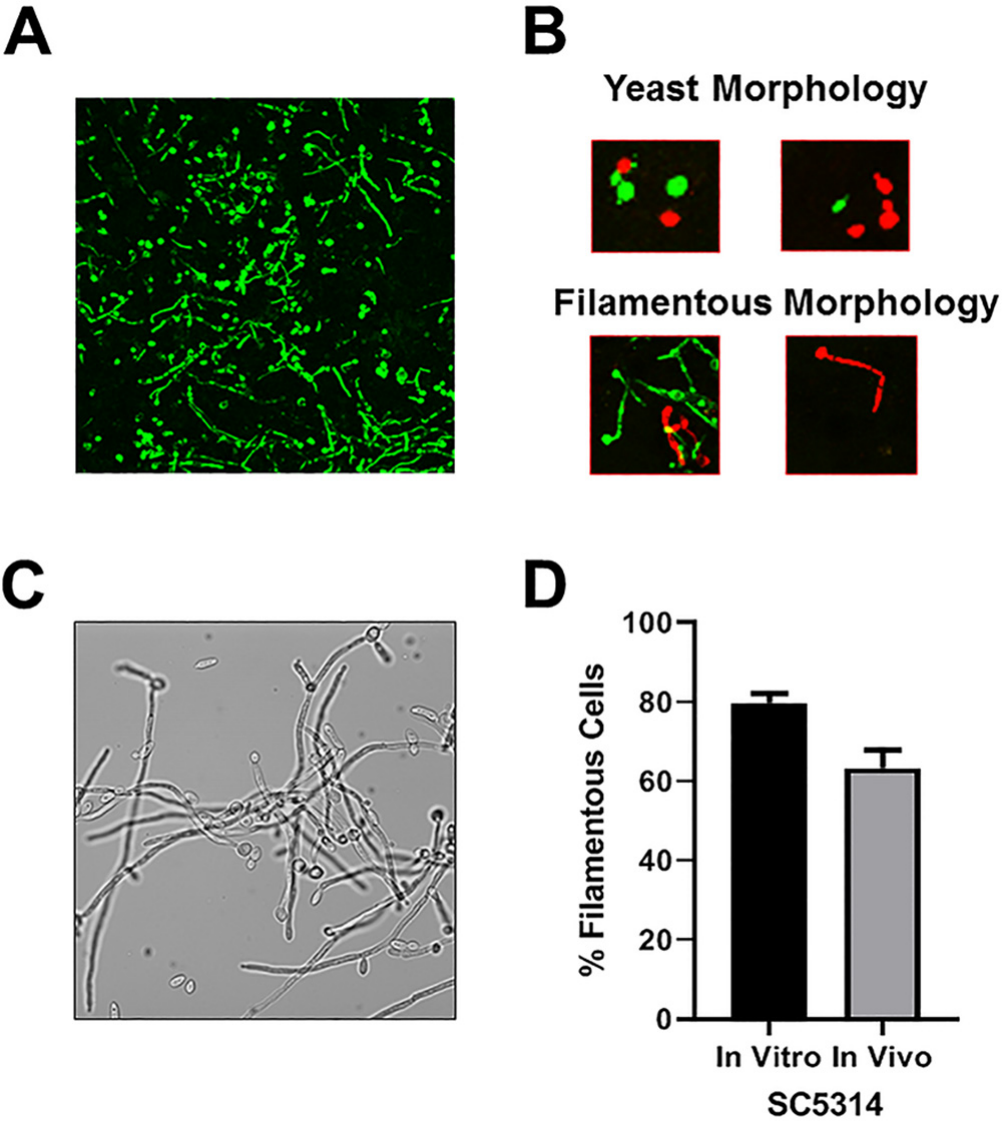
Comparison of filamentation of reference strain SC5314 *in vitro* and *in vivo* using the intravital imaging approach. (A) Representative field showing NEON-labeled SC5314 within tissue of the ear at 24 h postinfection. (B) Examples of yeast and filamentous morphologies as captured by intravital imaging assay. (C) SC5314 cells exposed to RPMI + 10% serum at 37°C for 4 h. (D) Comparison of the percentage of filamentous cells *in vitro* (RPMI + 10% serum at 37°C for 4 h) and *in vivo* (24 h postinfection). Bars indicate the mean of 4 to 5 fields from replicate experiments, with error bars indicating standard deviation.

To characterize the correlation between *in vitro* and *in vivo* filamentation phenotypes, we first constructed a NEON-labeled derivative of the standard reference strain SC5314 and injected it into the ears of DBA/2 mice (10); this strain of mice lacks complement C5, which limits initial edema due to reduced influx of phagocytes and thereby improves resolution. SC5314 undergoes robust filamentation in this model at 24 h postinfection (Fig. 2A). Although we can clearly distinguish yeast cells from filamentous cells (Fig. 2B), we are not able to consistently distinguish between hyphae and pseudohyphae and thus score filaments as “not-yeast” (see the Materials and Methods section for a complete description of scoring method). Thus, non-yeast cells represent filaments or filamentous cells and we will refer to them as such in the text. We also induced hyphae formation *in vitro* using RPMI medium supplemented with 10% bovine serum (Fig. 2C) for 4 h. For SC5314, comparable numbers of filaments are observed at 24 h *in vivo* and after 4 h of *in vitro* induction (Fig. 2D). This is consistent with the general observations in the literature indicating that SC5314 forms robust filaments under both liquid and plate-based conditions (5, 13).

**FIG 3:**
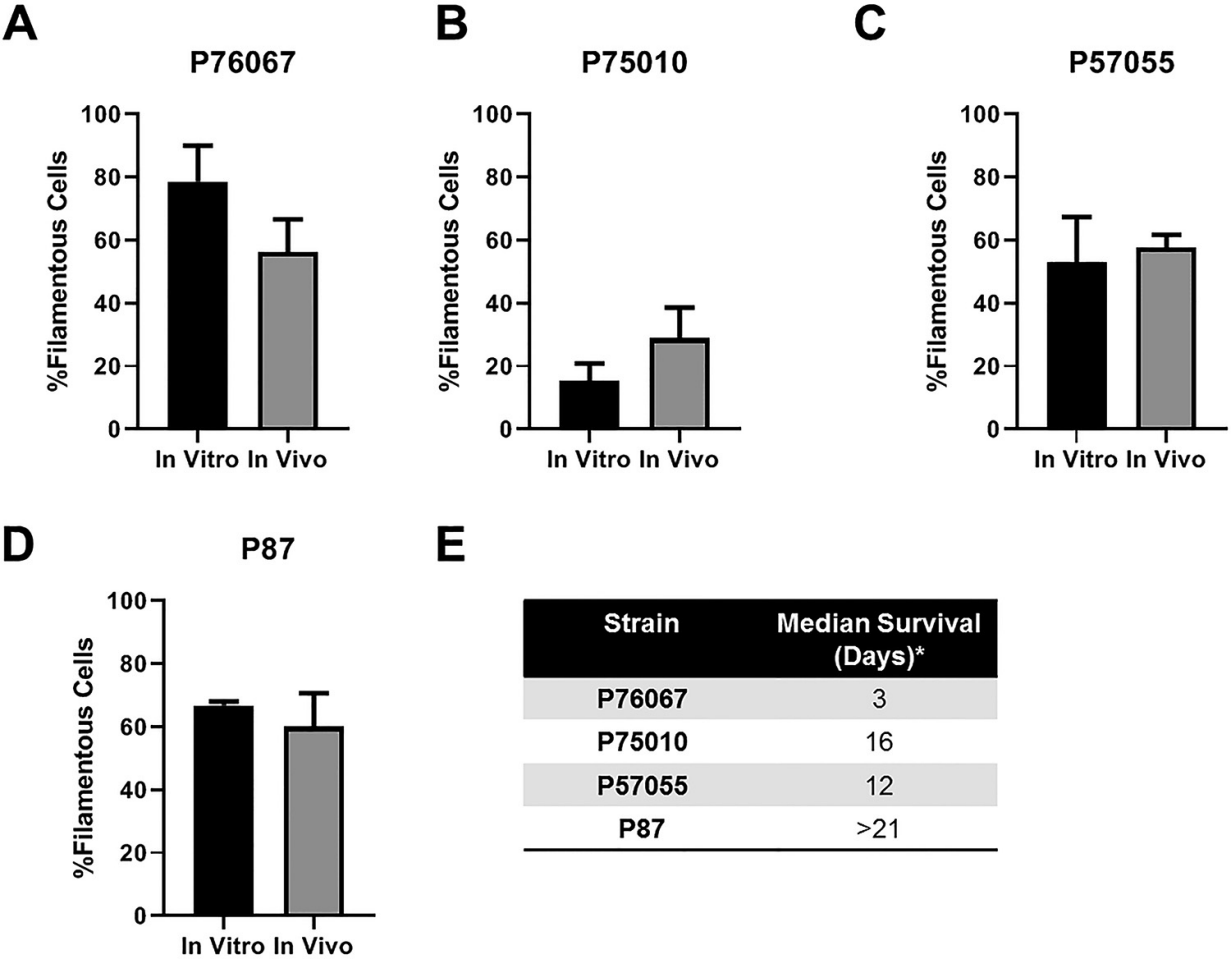
Comparison of *in vitro* and *in vivo* filamentation across four well-characterized clinical isolates of C. albicans. (A to D) The indicated isolates were imaged after exposure to RPMI + 10% serum at 37°C for 4 h (*in vitro*) or at 24 h postinjection into the ears of mice (*in vivo*). Bars indicate the mean of 4 to 5 fields from replicate experiments, with error bars indicating standard deviation. (E) Median survival of the clinical isolates calculated from data reported by Wu et al. (14).

To extend this analysis to strains with heterogenous filamentation phenotypes, we took advantage of the recent characterization of strains from a set of 21 clinical isolates that had also previously been characterized for virulence phenotypes (14, 15). We chose four strains (P87, P57010, P57055, and P76067) from different clades that had relative filamentation scores on solid Spider medium of P87∼SC5314∼P76067≫P57055∼P75010, while in liquid RPMI without serum the relative filamentation was: SC5314∼P87>P76067≫P57055∼P75010 (5, 13). The strains were engineered to express a fluorescent protein (Eno1-NEON) and assessed both *in vitro* and *in vivo* as described for the reference strain SC5314 (Fig. 3A and B). The addition of serum to RPMI medium induced greater filamentation of P57055 relative to RPMI alone (∼5% to 65%); otherwise, the relative order of *in vitro* filamentation phenotypes was similar to RPMI alone. However, the strain with the lowest amount of filamentation *in vitro* (P75010) formed 3-fold more filaments in the presence of serum relative to the absence, based on the data from Hirakawa et al. (14).

*In vivo*, three of the strains (P87, P76067, and P57055) filamented to similar extents and matched well with SC5314. Importantly, however, the strain that formed the least amounts of filaments (P75010) only differed by ∼2-fold from the other strains. Thus, the overall variation in filamentation phenotypes *in vivo* was less than observed *in vitro*, particularly with respect to the filamentation scores on solid medium (14). Indeed, the relative order of filamentation in RPMI + 10% serum matched that seen *in vivo* much better than solid Spider medium and slightly better than RPMI alone (13). Hirakawa et al. (14) and Azadmanesh et al. (6) had examined whether *in vitro* filamentation of these clinical strains or mutants correlated with virulence but had found no clear relationship. The four clinical isolates we examined have very different median survival rates, as was reported by Wu et al. (15) (Fig. 3E). For example, P87, which filamented well under all three *in vitro* conditions and *in vivo*, was the least virulent strain with no definable time to 50% survival. In addition, the variation in the virulence phenotypes reported for the other three strains is much wider than the variation in their relative abilities to filament *in vivo*. Additional studies of clinical isolates will be needed to establish a well-powered correlation between *in vivo* filamentation and virulence.

### Validation of a dual fluorophore assay to assess the effect of mutations on C. albicans filamentation during infection.

**FIG 4:**
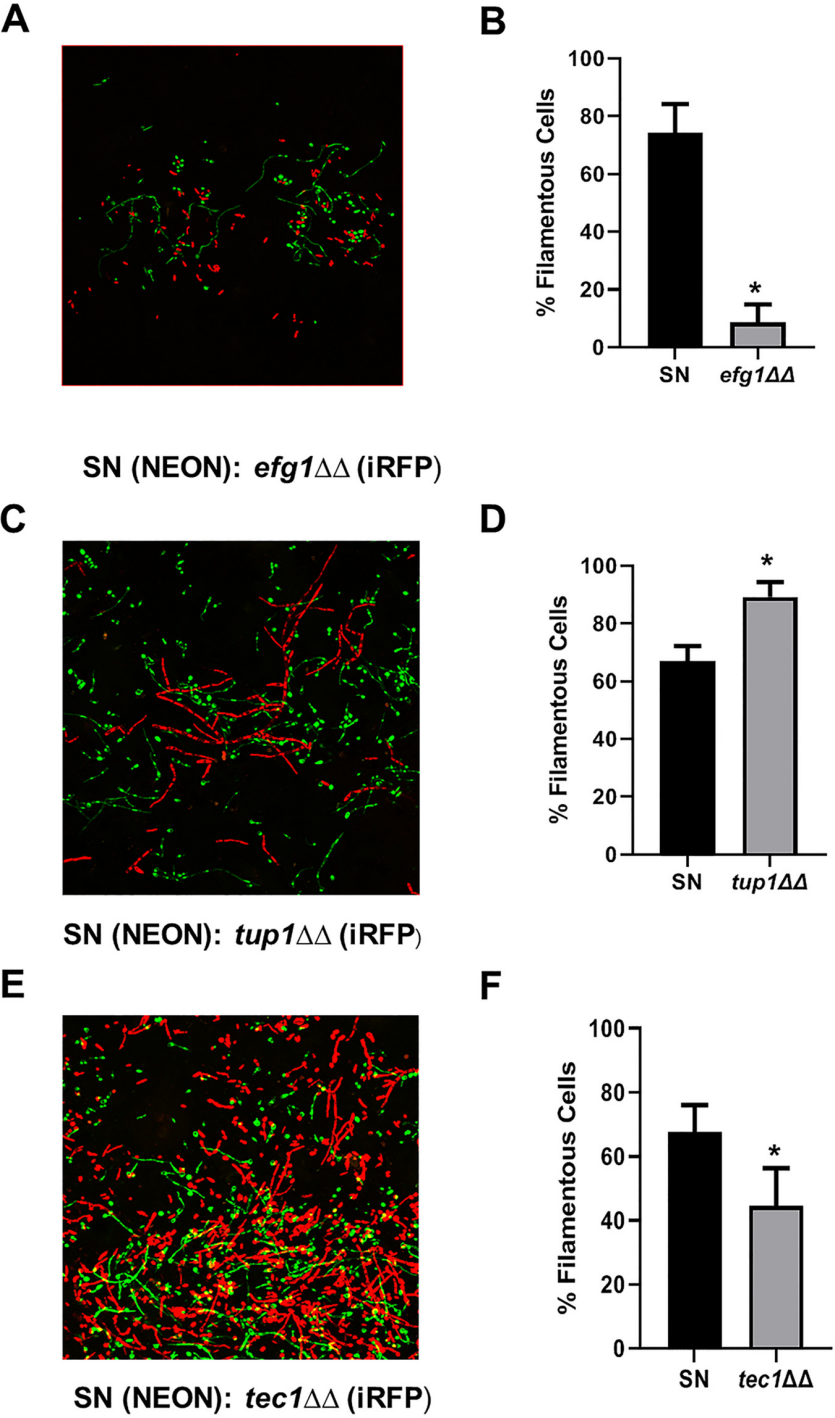
Validation of WT:mutant mixed infection model to assess effects of transcription factor deletion strains on *in vivo* filamentation. (A, C, and E) Representative fields for 1:1 WT(NEON):TF deletion mutant (iRFP) infections after 24 h postinfection with a 1:1 mixture of the indicated strains. (B, D, and F) Bars indicate the mean of 4 to 5 fields from replicate experiments with error bars indicating standard deviation. An asterisk (*) indicates *P* < 0.01 for a Student’s *t* test comparing the WT filamentation ratio to the indicated TF mutant.

The transcriptional regulation of C. albicans filamentation has been the subject of extensive study (3–7) and has led to the identification of a set of transcription factors (TFs) that play a role in filamentation. Previously, we and others have shown that this network of TFs appears to be dependent upon the specific environmental context for the filamentation (6, 7). Therefore, we hypothesized that the transcriptional regulation of filamentation during infection may have distinct patterns relative to *in vitro* conditions. To test this hypothesis, we applied our intravital imaging assay to the characterization of the ability of different TF deletion strains to undergo filamentation *in vivo*. In order to directly compare a given mutant to a reference control strain, we infected animals with an inoculum containing a 1:1 ratio of a reference strain (SN background) expressing *ENO1* fused with NEON and a homozygous *EFG1* deletion mutant derived from that reference strain containing *ENO1* fused with iRFP. As shown in Fig. 4A and B, *EFG1* is required for filamentation under both *in vitro* and *in vivo* conditions. Next, we examined a strain that is constitutively filamentous *in vitro* due to deletion of a transcriptional repressor of filamentation, *TUP1* (16). Hyper-filamentous strains are difficult to study using the intravenous inoculation model because they fail to establish infection. Consistent with its *in vitro* phenotype, only filamentous forms of the *tup1*ΔΔ mutant were observable both *in vivo* and *in vitro* (Fig. 4C and D).

Finally, we tested the ability of a strain lacking *TEC1* to filament *in vivo*. Tec1 is regulated by Efg1 and Cph2 *in vitro* and is required for full virulence (17, 18). Based on histological sections of mouse kidneys infected with a *tec1*ΔΔ strain, it appears that this strain retains the ability to filament *in vivo* despite being deficient in almost all *in vitro* conditions reported. As shown in Fig. 4E and F, the *tec1*ΔΔ strain forms filaments *in vivo* but the ratio of filaments to yeast is reduced relative to the reference strain (*P = *0.003, Student’s *t* test). It is possible that the coinfection of two strains could lead to results that are distinct from mono-strain infections. To test this, we compared the number of filamentous cells observed in a mono-strain infection with *tec1*ΔΔ to the number observed in a dual-strain infection of WT and *tec1*ΔΔ. The percentage of filamentous cells in the single strain infection did not differ significantly from the dual infection (46.8% ± 11.6% versus 35.8% ± 12.0%, *P* = 0.17, Student’s *t* test). This does not rule out the possibility of *trans*-effects for all strains, but suggests that such effects are not likely to be general. These experiments confirm that the assay can identify both hypo- and hyperfilamentous mutant strains. The discordant phenotype previously reported for *in vitro* and *in vivo* filamentation phenotypes for the *tec1*ΔΔ strain is recapitulated in our model and further suggests that inducers of filamentation in the stromal tissue of the ear may be similar to those operative in the kidney.

### Efg1 and Brg1 mutations reduce *in vivo* filamentation in C. albicans clinical isolates.

**FIG 5:**
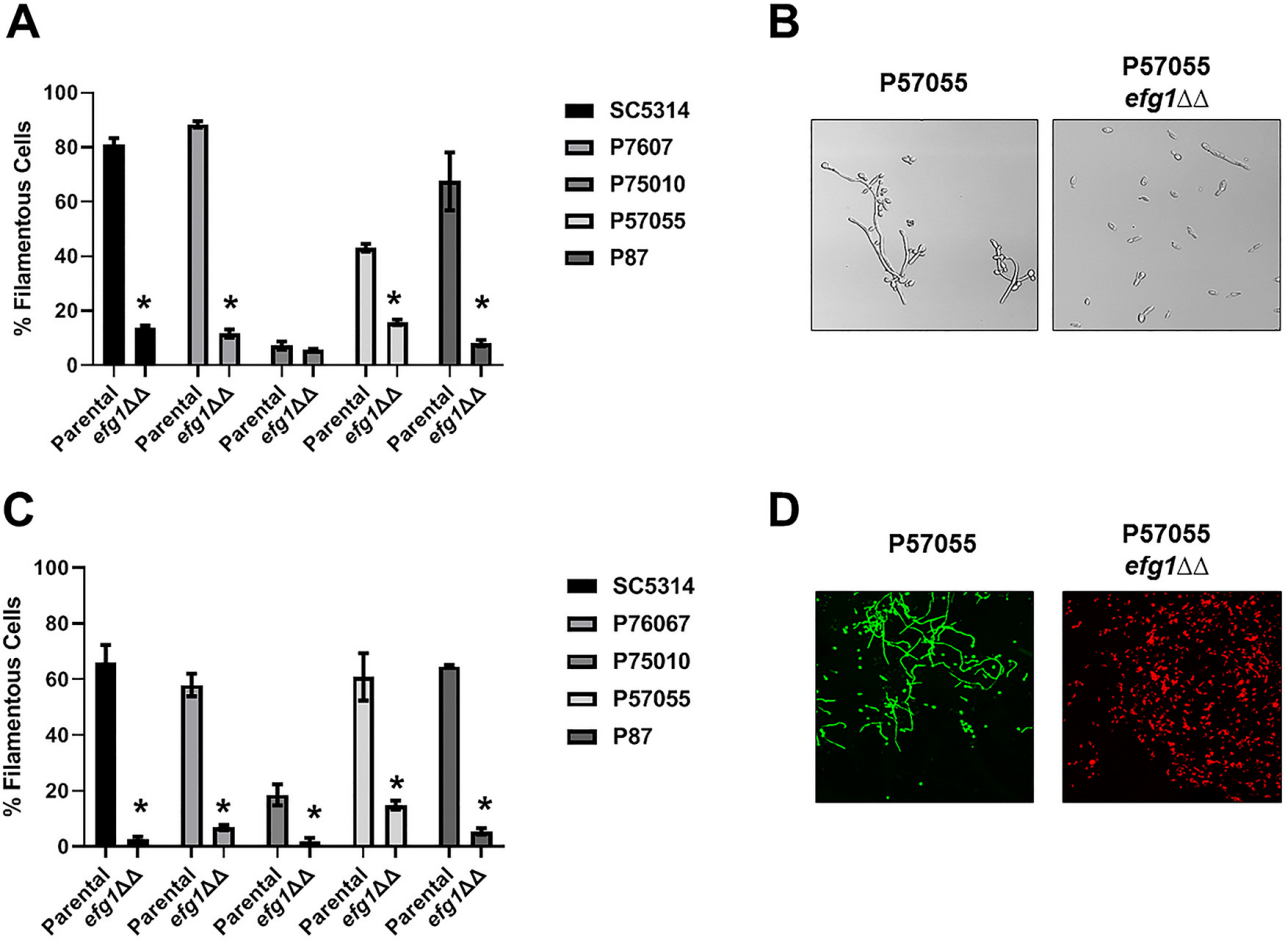
Efg1 is required for *in vitro* and *in vivo* filamentation across multiple C. albicans isolates. (A) Comparison of the *in vitro* filamentation (RPMI + 10% serum at 37°C for 4 h) of the parental and *efg1*ΔΔ strains derived from SC5314 and the indicated clinical isolates. Bars indicate the mean of 4 to 5 fields from replicate experiments, with error bars indicating standard deviation. Bars marked with an asterisk (*) indicate that the *efg1*ΔΔ mutant is statistically significantly different from the parental strain (*P* < 0.05; Student’s *t* test). (B) Representative images of *in vitro* filamentation for the parental and *efg1*ΔΔ derivative of P57055. (C) Comparison of the *in vivo* filamentation (24 h postinfection) of the parental and *efg1*ΔΔ strains derived from SC5314 and the indicated clinical isolates. Bars indicate the mean of 4 to 5 fields from replicate experiments, with error bars indicating standard deviation. (D) Representative images of *in vivo* filamentation for the parental and *efg1*ΔΔ derivative of P57055.

Once we validated the ability of the *in vivo* imaging assay to characterize mutants with both hypo- and hyperfilamentation phenotypes, we examined the effect of deleting master regulatory TFs in the five strains characterized above (5). Efg1 is one of the most widely studied transcriptional regulators of C. albicans and has been shown to be required for filamentation under both *in vitro* and *in vivo* conditions (19). Huang et al. found that Efg1 was critical to biofilm formation and *in vitro* filamentation in all five of the strain backgrounds (5). To extend our finding that it is required for *in vivo* filamentation in the SN genetic background, we labeled *efg1*ΔΔ mutants in SC5314, P87, P57010, P57055, and P76067 strains and tested each strain’s ability to filament in our standard *in vitro* conditions and *in vivo*. *In vitro*, deletion of *EFG1* reduced filamentation in all strains except P75010, which formed very few filaments at baseline (Fig. 5A and B); these data were similar to those previously reported by Huang et al. (5). Similarly, *efg1*ΔΔ mutants were significantly impaired for filamentation *in vivo*, with only mutants in the P57050 background forming more than 10% filaments (Fig. 5C and D).

**FIG 6:**
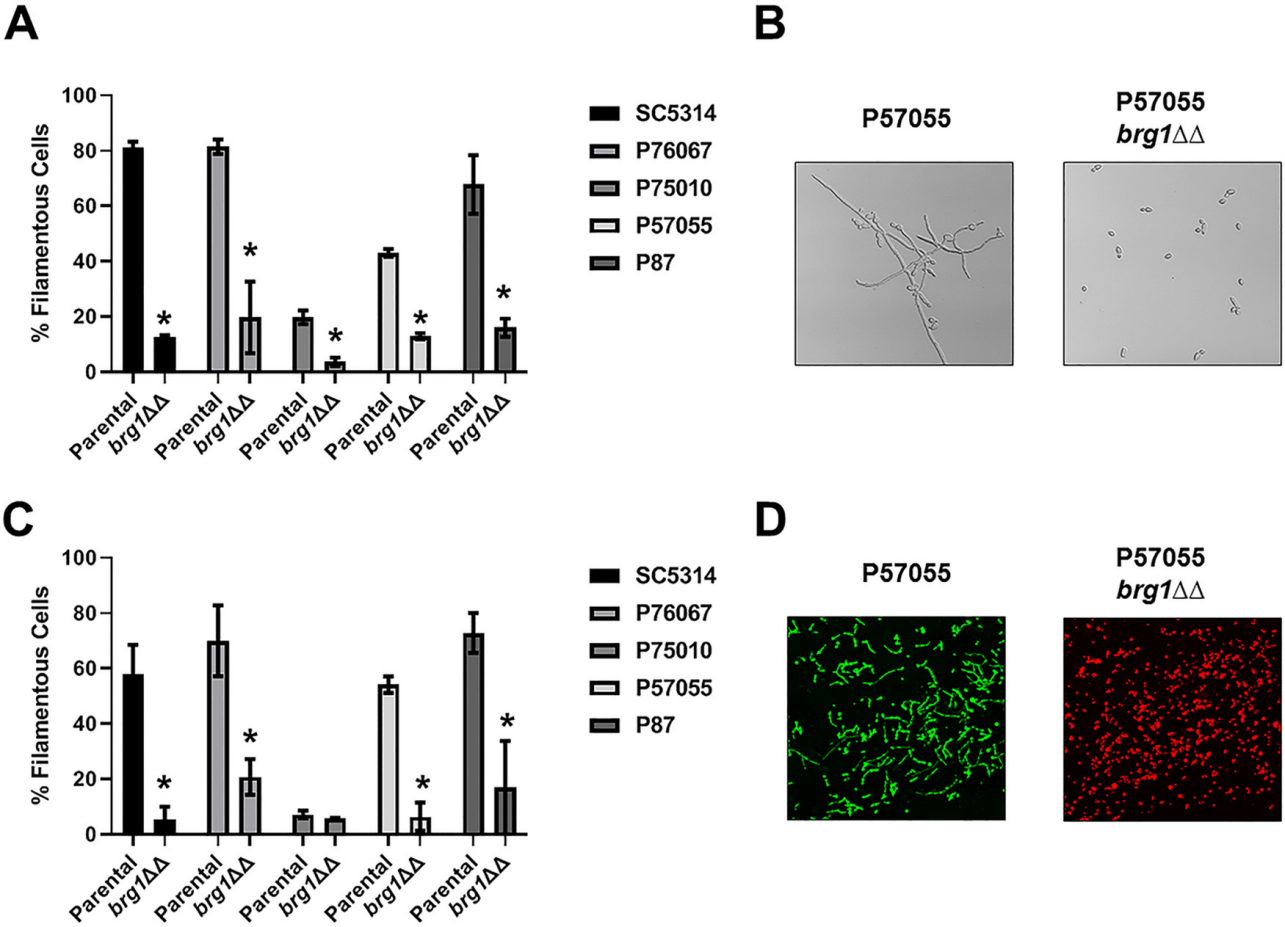
Brg1 is required for *in vitro* and *in vivo* filamentation across multiple C. albicans isolates. (A) Comparison of the *in vitro* filamentation (RPMI + 10% serum at 37°C for 4 h) of the parental and *brg1*ΔΔ strains derived from SC5314 and the indicated clinical isolates. Bars indicate the mean of 4 to 5 fields from replicate experiments, with error bars indicating standard deviation. Bars marked with an asterisk (*) indicate the *brg1*ΔΔ mutant is statistically significantly different from the parental strain (*P* < 0.05; Student’s *t* test). (B) Representative images of *in vitro* filamentation for the parental and *brg1*ΔΔ derivative of P57055. (C) Comparison of the *in vivo* filamentation (24 h postinfection) of the parental and *brg1*ΔΔ strains derived from SC5314 and the indicated clinical isolates. Bars indicate the mean of 4 to 5 fields from replicate experiments, with error bars indicating standard deviation. (D) Representative images of *in vivo* filamentation for the parental and *brg1*ΔΔ derivative of P57055.

The TF Brg1 also plays an important role in the regulation of filamentation through a feedback loop with Nrg1, a repressor of filamentation (20, 21). Huang et al. found that deletion of *BRG1* reduced filamentation in all isolates *in vitro* (5) and we observed similar results *in vitro* (Fig. 6A and B). *In vivo*, *brg1*ΔΔ mutants were uniformly deficient in filamentation by at least 5-fold relative to the parental strain (Fig. 6C and D). These observations indicate that the filamentation master regulator status of Efg1 and Brg1 TFs is retained during filamentation *in vivo*.

### Ume6 plays a modest role during *in vivo* filamentation.

**FIG 7:**
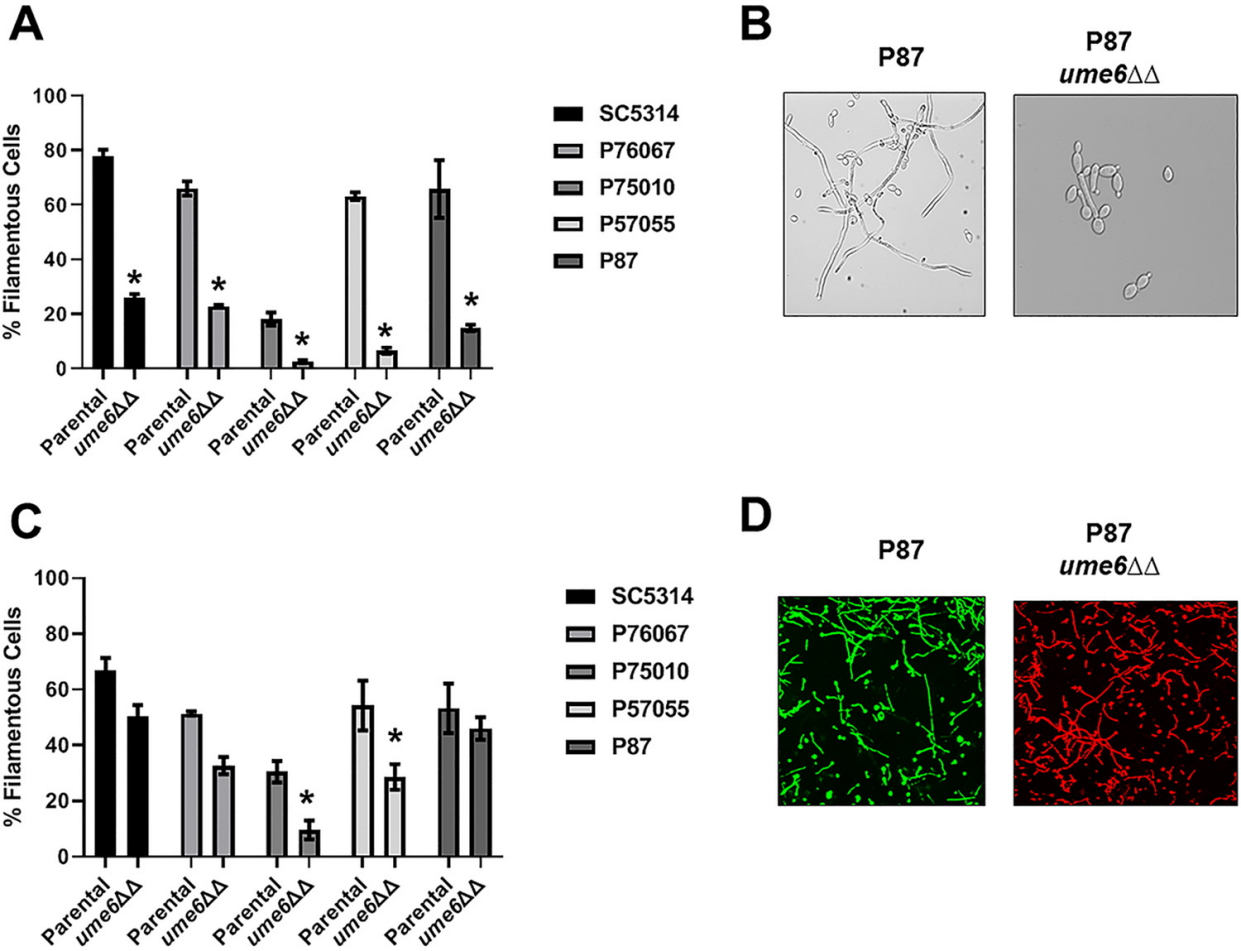
Ume6 has a more profound effect on filamentation *in vitro* than *in vivo*. (A) Comparison of the *in vitro* filamentation (RPMI + 10% serum at 37°C for 4 h) of the parental and *ume6*ΔΔ strains derived from SC5314 and the indicated clinical isolates. Bars indicate the mean of 4 to 5 fields from replicate experiments, with error bars indicating standard deviation. Bars marked with an asterisk (*) indicate that the *ume6*ΔΔ mutant is statistically significantly different from the parental strain (*P* < 0.05; Student’s *t* test). (B) Representative images of *in vitro* filamentation for the parental and *ume6*ΔΔ derivative of P87. (C) Comparison of the *in vivo* filamentation (24 h postinfection) of the parental and *ume6*ΔΔ strains derived from SC5314 and the indicated clinical isolates. Bars indicate the mean of 4 to 5 fields from replicate experiments, with error bars indicating standard deviation. (D) Representative images of *in vivo* filamentation for the parental and *ume6*ΔΔ derivative of P87.

*In vitro*, Ume6 is a well-characterized transcriptional regulator of filamentation whose expression has been shown to be necessary and sufficient to drive this process (22). Consistent with this role, deletion of *UME6* reduces filamentation 3- to 4-fold under *in vitro* conditions for all five strains (Fig. 7A and B). Under *in vivo* conditions, however, this level of reduction in filamentation was only seen in the *ume6*ΔΔ strain derived from P75010, the poorest filamenting strain (Fig. 7C and D). Deletion of *UME6* reduces filamentation by less than 1.5-fold for SC5314, P76067, and P87, while having a 2-fold effect on P57055; there was essentially no difference between the filamentation of P87 and its *ume6*ΔΔ mutant. Thus, most strains formed significant numbers of filaments *in vivo* in the absence of *UME6*. These observations indicate that the *in vivo* stimuli that lead to filamentation *in vivo* must trigger this process in a manner that largely bypasses the function of Ume6. Since Ume6 is required for filamentation under a variety of *in vitro* conditions (23), our data strongly support the hypothesis that the transcriptional networks for C. albicans vary with the specific environmental context.

### Bcr1 is dispensable for filamentation *in vivo*.

**FIG 8:**
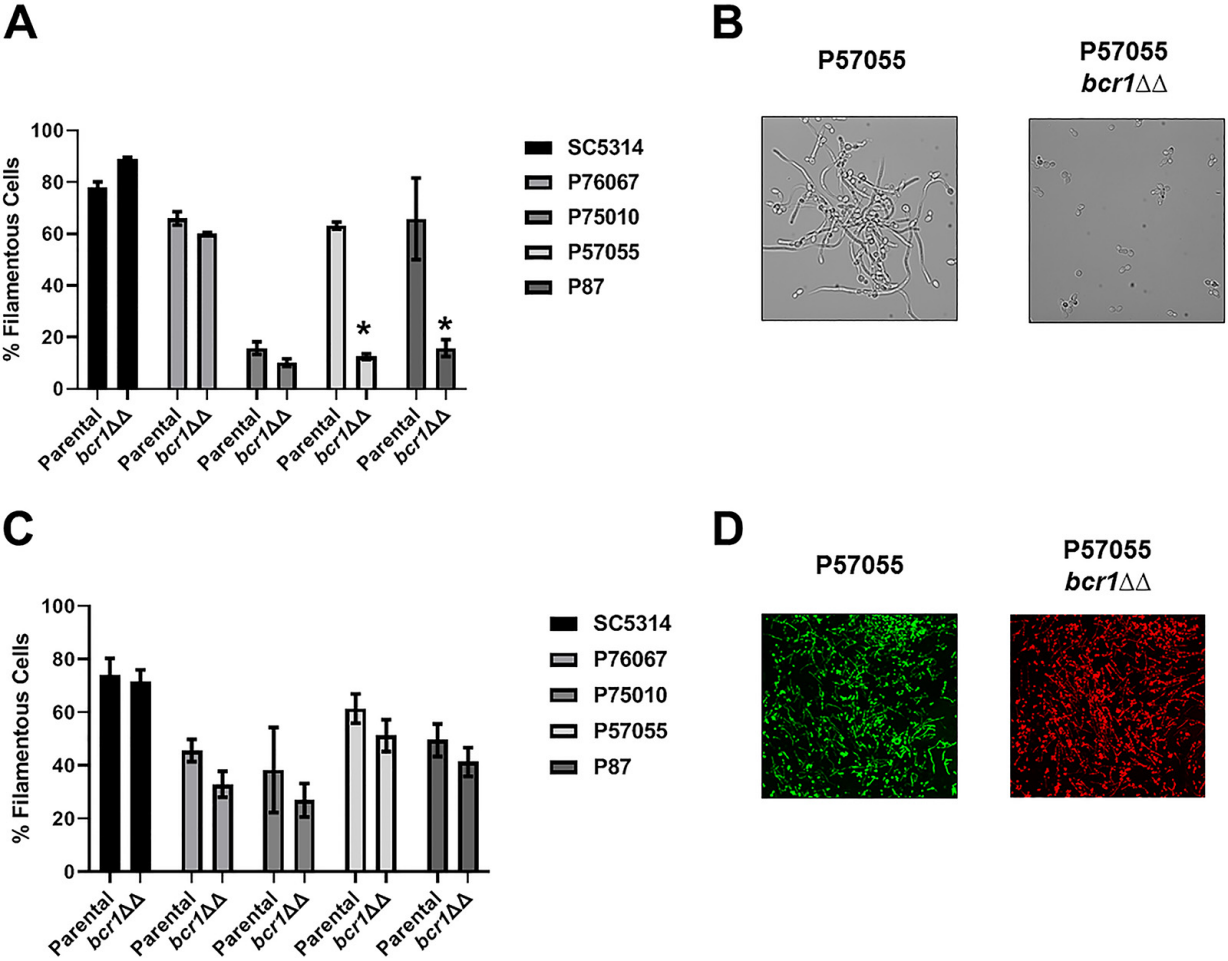
Bcr1 is dispensable for filamentation *in vivo*. (A) Comparison of the *in vitro* filamentation (RPMI + 10% serum at 37°C for 4 h) of the parental and *bcr1*ΔΔ strains derived from SC5314 and the indicated clinical isolates. Bars indicate the mean of 4 to 5 fields from replicate experiments, with error bars indicating standard deviation. Bars marked with an asterisk (*) indicate that the *bcr1*ΔΔ mutant is statistically significantly different from the parental strain (*P* < 0.05; Student’s *t* test). (B) Representative images of *in vitro* filamentation for the parental and *bcr1*ΔΔ derivative of P57055. (C) Comparison of the *in vivo* filamentation (24 h postinfection) of the parental and *bcr1*ΔΔ strains derived from SC5314 and the indicated clinical isolates. Bars indicate the mean of 4 to 5 fields from replicate experiments, with error bars indicating standard deviation. (D) Representative images of *in vivo* filamentation for the parental and *bcr1*ΔΔ derivative of P57055.

Bcr1 is a critical regulator of gene expression during biofilm formation both *in vitro* and *in vivo* (24, 25). Bcr1 has not typically been associated with the regulation of *in vitro* filamentation (4, 7), although it has been reported to negatively regulate the filamentation of opaque cells *in vitro* (26). Huang et al., however, found that Bcr1 regulated *in vitro* filamentation in P57055 and P87 but not in SC5314 or P76067 (5); our *in vitro* data matched those findings (Fig. 8A and B). For strains for which *in vitro* filamentation was dependent on *BCR1*, Huang et al. also found that expression of *BRG1* was dependent on *BCR1* (5). *In vivo*, however, the deletion of *BCR1* had a minimal effect on the filamentation of any of the strains with the mutant forming filaments at a rate within 15% of the parental strain. Thus, *in vivo* filamentation is not dependent on the Bcr1-Brg1 interaction even in strains for which this regulatory circuit is required for filamentation *in vitro*. These observations further support the hypothesis that distinct transcriptional circuits regulate *in vitro* and *in vivo* filamentation.

## DISCUSSION

New approaches to directly characterizing virulence-associated phenotypes and mechanisms using mammalian infection models will be needed to advance the study and understanding of C. albicans pathogenesis (9, 10). With this goal in mind, we have developed an intravital imaging strategy that allows the characterization of filamentation in an anatomic site that is relevant to the infection process. We have also taken advantage of the recent realization that the study of clinical isolates with diverse ranges of phenotypes can provide important information not readily available by the study of laboratory reference strains (5, 14). These experiments have allowed us to make three major conclusions regarding the relationship between C. albicans filamentation *in vitro* and *in vivo*, as discussed below.

Before we discuss these conclusions, it is important to consider how this model integrates with previous approaches to the assessment of C. albicans filamentation *in vivo*. For example, a potential limitation of this approach is that the anatomic site of the infection may not be representative of other sites. However, our results for TFs such as *TEC1* and *UME6* correlate with previously reported kidney histology for these deletion strains (18, 23), suggesting there is significant overlap. However, it is important to note that *EFG1*, a canonical master regulator of filamentation, is not required for filamentation in the oral cavity of gnotobiotic pigs (27) and its deletion mutant is hyperfilamentous under *in vitro* embedded conditions (28). Furthermore, Witchley et al. have reported that *ume6*ΔΔ deletion mutants in the SN background filament similarly to wild-type (WT) cells in a model of commensal colonization of the GI tract (9). They also found that the *tec1*ΔΔ mutant was similar to wild type while *efg1*ΔΔ and *brg1*ΔΔ strains formed predominantly yeast. Our data are very similar to their findings indicating that Efg1 and Brg1 are important regulators of filamentation in multiple niches and that Ume6 plays a modest role in these niches. Thus, the phenotypes that we observe for these well-studied filamentation-related TFs are reasonably concordant with other examples of *in vivo* assessments of filamentation. The method of Witchley et al. (9) is a commensal counterpart to our approach in that both provide quantitative data in real time or near real time. One interpretation of our results in context with these other reports is that it seems likely that the transcriptional regulation of C. albicans filamentation varies from one niche to another. As such, genes required for filamentation in kidney, the most studied target organ in mice, may be different from the oral cavity, the submucosal stroma, or the mucosa of the GI tract.

The choice of mouse strain warrants some discussion. As indicated above, we used DBA/2 mice to limit the influx of inflammatory cells during the infection. This decision was based primarily on both technical considerations. Specifically, inflammation-induced edema reduces resolution and limits our ability to distinguish morphotypes. It is certainly possible that the reduced influx of inflammatory cells may reduce or, in principle, increase filamentation of some mutants, and thus our model will be limited in its ability to detect those mutants. Indeed, it is well-established that C. albicans undergoes robust filamentation in the phagolysosome of macrophages. As such, some clinical isolates may not filament robustly in the presence of the inducing factors present in the tissue but then show increased filamentation in the presence of inflammatory cells or changes in tissue oxygenation that accompany damage to the sub-epithelium. With these caveats in mind, we feel the model provides a convenient approach to characterizing the relative abilities of strains and mutants to undergo filamentation during infection of mammalian tissue.

**FIG 9:**
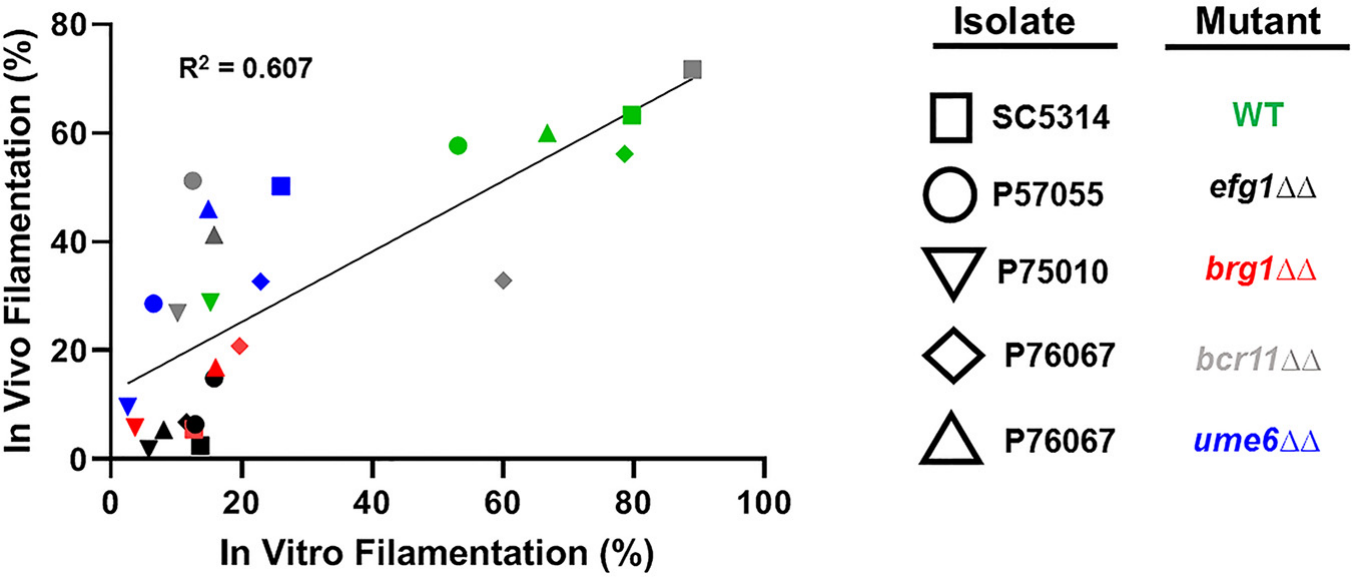
Summary of *in vitro* and *in vivo* comparison of filamentation for each strain and mutant tested in this study. The mean percentage filamentation for each strain *in vivo* and *in vitro* is plotted. Linear regression was performed to assess the correlation between *in vivo* and *in vitro* filamentation for the set of strains. The *R*^2^ value is shown. The specific strains can be identified using the shape (isolate) and color (mutant) as indicated by the tables in the figure.

The major findings from our study include, first, that by characterizing the *in vivo* filamentation of a laboratory reference strain (SC5314) and four clinical isolates with distinct virulence and *in vitro* filamentation phenotypes, we demonstrated that the relative abilities of the strains to filament *in vivo* correlate to some extent with *in vitro* filamentation in RPMI supplemented with 10% serum. This is summarized in Fig. 9, where we have plotted the percentage of filamentous cells *in vitro* and *in vivo* for each parental strain and TF mutant examined in our study. The correlation between *in vitro* and *in vivo* filamentation was moderate (*R*^2^ = 0.607) with a slope less than 1; however, this correlation seems to be mainly driven by the high- and low-filamenting strains. Many of the mutants and strains had significantly discordant filamentation ratios when comparing *in vitro* and *in vivo* experiments.

Although it is not possible to directly correlate the semiquantitative plate-based colony assays reported by Hirakawa et al. to our quantitative *in vivo* data (14), we found that one of the poorest filamenting strains (P57055) based on plate assays filamented quiet well in both RPMI + 10% serum and *in vivo*. Not surprisingly, these results suggest that C. albicans is subject to signals that induce filamentation *in vivo* that are not replicated, either in type or extent, *in vitro*. Although not wholly unexpected based on recent studies demonstrating that C. albicans mutant strains vary in their *in vitro* filamentation phenotypes based on the specific induction conditions (6, 7), our experiments are the first to directly compare *in vivo* and *in vitro* filamentation phenotypes and make this observation. More strains will need to be examined to determine robustness of the general correlation between RPMI supplemented with serum and *in vivo* filamentation. It appears that C. albicans filamentation within colonies on agar plates is relatively distinct from that occurring within mammalian tissue (6).

We note that Tucey et al. recently reported filamentation data for the same set of strains during *ex vivo* infection of bone marrow-derived macrophages (29). Consistent with our *in vivo* and *in vitro* studies, P87 and SC5314 showed a high hyphal index. P75010 essentially formed no hyphae under these conditions, while P76067 and P57055 also had low hyphal indices (29). The worst and best filamenting strains correlated well with our *in vivo* studies, while the intermediate strains filamented much better *in vivo* than during macrophage infection. Tucey et al. found that strongly filamenting strains triggered NLRP3-mediated pyroptosis, while those strains with lower hyphal indices did not (29). It would be interesting to determine if these strains filament more robustly in macrophages within the host. Taken together, it appears that some strains of C. albicans form filaments robustly under most inducing conditions, while others require more specific conditions. Consequently, one should be cautious when making very general or absolute statements regarding the role of a strain or mutant in filamentation based observations made *in vitro* or *ex vivo*, particularly if only a few conditions are tested.

Our second major finding was that the least virulent strain, P87, was able to filament strongly *in vivo* and *in vitro*. We also observed that the variation in *in vivo* filamentation among the five strains we studied was much less than the variation in virulence (15). Hirakawa et al. were unable to correlate *in vitro* filamentation with the virulence of these strains (14) and our data extend that lack of correlation to *in vivo* filamentation as well. *In vitro*, this lack of correlation was driven by the fact that seemingly non-filamentous strains such as P75010 are nonetheless virulent to a considerable degree (15). Our data suggest that this lack of correlation may be due instead to the fact that strains with very different virulence phenotypes all form a significant number of filaments *in vivo* (at least 25 to 30%). Previously, Noble et al. reported that *in vitro* filamentation and the ability to establish infection were not well correlated in large-scale pooled infectivity screens (30). As such, our data provide additional support for the notion that factors beyond filamentation are likely to contribute to the ability of C. albicans to cause disease, and that these factors vary in expression among clinical isolates.

Our third major finding was that, by studying a set of TF deletion mutants in the different C. albicans clinical strains, we have found that the function of specific TFs and TF circuits vary between *in vivo* and *in vitro* filamentation. Although Efg1 and Brg1 appear to retain their key roles regulating filamentation *in vitro* and *in vivo*, our observations suggest that the role of Ume6 is relatively modest *in vivo*. Under *in vitro* conditions, our data were consistent with previous reports that *UME6* deletion mutants have significant filamentation defects (22, 23). In the strongly filamenting strains SC5314 and P87, *ume6*ΔΔ mutants, the number and general quality of the filaments appeared to be quite similar to the parental strains. Indeed, the extent of filamentation observed for *ume6*ΔΔ mutants of SC5314 and P87 is very similar to histological sections of mouse kidney infected with a strain in which expression of *UME6* was transcriptionally repressed (23). The correlation of the *ume6*ΔΔ phenotypes with kidney histology further validates the ear infection model as representative of *in vivo* C. albicans filamentation. Overexpression of *UME6* increases filamentation and increases virulence and is clearly important for *in vitro* filamentation. However, it appears that *in vivo* signals that stimulate filamentation do so in a manner that is largely independent of Ume6. We also found that the Bcr1-Brg1 circuit, which is critical for *in vitro* filamentation and biofilm formation in some clinical strains (5), was not operative *in vivo*. These observations provide strong evidence that although C. albicans filamentation is a central part of its pathobiology, the TFs and transcriptional networks that regulate filamentation vary with the specific environmental cues such that even critical regulators of this process *in vitro* can be bypassed *in vivo*.

Taken together, our data provide strong support for the notion there are diverse regulatory mechanisms behind the complex phenotype of C. albicans filamentation and that these mechanisms vary with the specific niche or environmental context.

## MATERIALS AND METHODS

### Strains, cultivation conditions, and media.

The C. albicans clinical isolate strains and their respective mutants, as well as the SN background-derived TF deletion mutants, have been described previously (5, 7). All Candida albicans strains were precultured overnight in yeast peptone dextrose (YPD) medium at 30^°^C. Standard recipes were used to prepare media (4). RPMI medium was purchased and supplemented with bovine serum (10% vol/vol).

### Strain construction.

Fluorescently labeled strains were generated by using p*ENO1*-*NEON*-*NAT1* and *pENO1-iRFP-NAT1* plasmids (8, 31). All transcription factor mutants were tagged with iRFP and their respective parent strains were tagged with green fluorescent protein (NEON). Briefly, the plasmids were digested with NotI enzyme for 2 h at 37˚C and, subsequently, the linearized plasmid was further inserted into the *ENO1* locus (8). The C. albicans transformation was performed using the standard lithium acetate transformation method (32) and the transformants were selected using nourseothricin resistance marker (200 μg/ml, NAT; Werner Bioagents, Jena, Germany).

### Preparation and inoculation of mice with C. albicans.

The mutants and their respective parent strains were grown overnight in YPD at 30˚C. Harvested cells were washed thrice with sterile phosphate-buffered saline (PBS) and counted with a hemocytometer. A 1:1 mixture of NEON- tagged reference strain and iRFP-tagged mutant strain was mixed to get a final count of 1 × 10^8^ CFU/ml in PBS. The 5- to 6-week-old female DBA2/N mice (Envigo) used in these experiments were maintained on chlorophyll-free chow to minimize endogenous fluorescence. Prior to injections, the mice were anesthetized with isoflurane using SomnoSuite low flow anesthesia machine (Kent Scientific) and the hair on the ears was removed by chemical depilation. Aliquots of 1 × 10^6^ CFU/ml (10 μl) of C. albicans cells containing equal volume of reference and mutant strains (1:1) were injected into the dorsal ear dermis of anesthetized mice with a 29G1/2 needle. A characteristic papule was observed at the site of injection, indicating a successful intradermal injection.

### Confocal fluorescence microscopy.

At 24 h postinjection, mice were anesthetized using isoflurane. Mice were placed on the stage in the supine posture permitting ventral side of the ear facing downward for the imaging (10). Confocal images were carried out with a multiphoton laser scanning microscope (SP8; Leica Microsystem). The NEON and iRFP were excited at 488 nm and 635 nm, respectively and emission was detected using 505 to 525 nm and 655 to 755 nm bandpass filters, respectively. The minimum of 30 z-stacks with an interslice interval between 0.57 μm was acquired with a 25× water immersive objective lens. The collected images were further max stacked using ImageJ software and used for analysis.

### Scoring criteria.

*In vitro* hyphae ratios were scored as previously described (7). *In vivo* filamentous cells had identifiable mother cells and the filamentous projection was at least twice the length of the mother cell body. Yeast cells were round and/or budded and cells were termed as such. Filamentous cells were quantified manually by following the hyphal projection through each z-stack (*n* = >100 cells). Yeast cells were further required not to project through multiple z-stacks. Statistical significance was determined by the unpaired Student's *t* test. The data sets did not show a detectable difference from normality using the Shapiro-Wilk test (*P > *0.05). Statistical tests were performed using GraphPad Prism software.

### *In vitro* hyphal induction.

C. albicans strains were incubated overnight in YPD at 30°C, harvested, and diluted into RPMI + 10% serum at a 1:50 ratio and incubated at 37°C for 4 h. Cells were collected and examined by light microscopy directly.
